# Experiences, concerns, and needs of pregnant and postpartum women during the Covid-19 pandemic in Cyprus: a cross-sectional study

**DOI:** 10.1186/s12884-022-05017-y

**Published:** 2022-09-05

**Authors:** Eleni Hadjigeorgiou, Paris Vogazianos, Maria-Dolores Christofi, Emma Motrico, Sara Domínguez-Salas, Ana R. Mesquita, Andri Christoforou

**Affiliations:** 1 Nursing Department, School of Health Sciences, Cyprus University of Technology, Limassol, Cyprus; 2 Department of Social and Behavioral Sciences, School of Humanities, Social and Education Sciences, European University Cyprus, Nicosia, Cyprus; 3 Psychology Department, Universidad Loyola Andalucia, Dos Hermanas Seville, Spain; 4 School of Psychology, University of Minho, Campus de Gualtar, Braga, Portugal

**Keywords:** COVID-19, Perinatal, Pregnant, Postpartum, Descriptive survey study

## Abstract

**Background:**

The current COVID-19 pandemic is a unique stressor with potentially challenging and negative consequences on the experiences of pregnant and postpartum women. International literature highlights the pandemic’s negative impact on women’s perinatal experiences. This is the first study in the scientific literature reporting on the impact of the COVID-19 pandemic, on the perinatal experiences of a large sample of women living in Cyprus.

**Aim:**

To examine the impact of the COVID-19 pandemic on the experiences, concerns and needs of pregnant and postpartum women in Cyprus.

**Method:**

The cross-sectional study was conducted from July 2020 to January 2021. A total of 695 women, 355 pregnant and 340 postpartum women (with infants up to 6 months of age), residing in Cyprus were surveyed.

**Results:**

The great majority of the participants (80.9%) perceived the impact of the COVID-19 pandemic on their life as negative. The greatest sources of stress were identified and quantified for their impact on the participants. Our findings indicate that 74.1% of the pregnant women were concerned about changes due to COVID-19 measures impacting the presence of their family at the time of delivery, 57.2% about their newborn’s health, and 43.1% about changes related to perinatal care. Postpartum women’s concerns were mainly related to the welfare and health of their child (70.3%), whilst half of them (49.1%) expressed concerns about how they were going to care for their baby because of pandemic-related changes. Qualitative data revealed emerging themes as the basis of the pregnant and postpartum women’s concerns and needs.

**Conclusions:**

The COVID-19 pandemic and the associated imposed measures and restrictions had adverse effects on pregnant and postpartum women’s perinatal experiences in Cyprus. The women’s concerns emphasized the need for the development of specialized, evidenced-based support systems which are essential particularly in pandemic-like situations, when pregnant and postpartum women are more vulnerable to isolation.

## Introduction

### Background

The COVID-19 pandemic and the associated state-imposed measures for the minimization of risk, and management of those affected by the virus have resulted in unprecedented changes in people’s lives [1]. A growing body of evidence illustrates how the COVID-19 pandemic and the associated public-health measures, affected global mental health [2]. Besides the direct threat posed by COVID-19 on physical health, challenges for mental health arise through indirect consequences of the pandemic [3]. Changes in people’s daily lives, including changes in employment, finances and childcare, along with state-imposed preventive measures (e.g. social distancing, self-quarantine, lockdowns, curfews, prohibition of travel and closing of borders in some instances) to reduce exposure to and spread of the virus have led to greater psychological distress, anxiety, stress and depression [4–8]. An investigation of mental health in Victoria, Australia - the region with the lowest SARS-CoV-2 prevalence globally - shows persistently common experiences of adverse mental health symptoms, suggesting that the impact of the pandemic on mental health may be oblivious to objective infection risk [9].

A less researched population – pregnant and postpartum women – appear to be more vulnerable to pandemic-related stressors compared to the general population, since the perinatal period (pregnancy and first year postpartum) is generally a time of high vulnerability to mental health problems [10]. Studies to date show an increase in the prevalence of depression and/or anxiety symptoms in perinatal women [11–14]. Risk factors were mainly related to the possibility of COVID-19 infection, changes in the organization of perinatal care, social isolation and financial problems [11–14]. Although more representative data are needed, a rise in the prevalence of domestic violence is also a possible risk factor [12].

### The case of Cyprus

SARS-CoV-2 first appeared in Cyprus on March 9, 2020 and was followed by immediate governmental response (i.e. restrictions in social gatherings, school closures, flight cancellations and travel bans) [15]. Over time, the Cypriot government imposed some of the stricter measures in the EU (i.e. quarantines, lockdowns, curfews, and other social distancing measures) in order to contain the spread of the virus and reduce the strain on the healthcare sector. Indicatively, during the first lockdown, which was imposed from March 24 until April 30, 2020 (initially planned until April 13, 2020), all movements were restricted, with the following exceptions: travelling to and from work (only for jobs requiring physical presence), purchase of goods and services deemed essential (e.g. civil services, supermarkets, pharmacies, doctors, banks), provision of assistance to vulnerable family members, attendance at crucial and significant religious ceremonies (e.g. funerals and major religious feasts), and physical exercise or dog walking within the immediate vicinity of the neighborhood. All citizens were required to send a text message or fill out a mandatory form for pre-approval of movement and to carry a valid form of identification when engaging in any of the aforementioned activities. Except for work, movement was restricted to a maximum of two times per day and for a limited amount of time. Parks, playgrounds, pedestrian areas, beaches and picnic areas, as well as places of worship were off limits. With few exceptions, the operation of retail businesses was suspended [16]. On March 30, 2020, even stricter restrictions on movement were imposed through a curfew from 21:00 to 06:00.

After a slowdown of COVID-19 cases in May 2020, the government began to ease the restrictions in phases. During the summer months of 2020, the daily number of cases were very few or sometimes zero. A significant increase of COVID-19 cases in October led to the re-introduction of emergency measures, which were followed by partial and targeted lockdowns, an island-wide curfew from 21:00 till 05:00 in November, and a mandate for working from home (imposed for the public sector and strongly advised for the private sector) at the end of December 2020 [15]. A second lockdown was announced for the period January 10 - January 31, 2021. Again, movement was restricted to twice a day and only under certain circumstances; retail businesses shut down and distance learning was re-introduced in schools, with the existing curfew remaining in force [17].

As in most EU member states, pregnancy and childbirth healthcare services and practices in Cyprus did not remain unaffected. In March 2020, for example, some women faced forced changes in the healthcare provisions they were entitled to, including forced changes to their consultant obstetricians and hospitals, due to the closure of maternity units in Paphos, Famagusta and Limassol General Hospitals. State Health Services Organization (SHSO) developed protocols in maternal and newborn care that focused exclusively on controlling and minimizing the risk of infection at all costs, often resulting in detrimental effects for evidence-based practice and adequate quality maternity care. According to the protocols developed and imposed by SHSO, the presence of spouses or partners was prohibited both during childbirth and during the mother’s stay at hospital, while pregnant women who tested positive for COVID-19 were required to give birth through a caesarean section and to be separated from their newborns shortly after birth [18–21]. These women were transferred to a designated unit in the Famagusta hospital and were not allowed to breastfeed, while newborns had to be isolated in separate rooms and bottle-fed by midwives. Women who wished to feed their babies with breastmilk needed to use breast pump kits and pump the milk whilst wearing surgical masks [22]. Privately-owned maternity clinics had largely implemented the measures specified in the protocols published by SHSO, with many adopting even stricter policies of their own accord [23].

Ethical concerns were raised by women’s groups and the non-governmental organization “Birth Forward”, as the measures were considered disproportionate to the risk of COVID-19 infection. Furthermore, concerns have also been raised over violation of women’s rights during childbirth, as well as the prohibition of evidence-based practices fostering the well-being of both mother and child, and the rest of the family (e.g. individualized mode of birth based on women’s preferences and obstetric indications, the presence of their spouse or partner or any other person during birth, skin-to-skin contact, rooming-in). These measures exacerbated the already existing problems and gaps in perinatal care in Cyprus, including the medicalization of pregnancy and birth, the very high rate of caesarean sections, and incidences of obstetric violence [24, 25]. On May 8, 2020, the Cyprus Commissioner for Administration and the Protection of Human Rights intervened with an own-initiative report, suggesting that maternity clinics in both public and private hospitals should allow fathers with a COVID-19 negative test to be present during childbirth, in accordance with the scientific recommendations provided by the WHO, the International Confederation of Midwives, and the United Nations Population Fund [26]. The Commissioner also emphasized the rights of expectant mothers for a safe and positive experience during childbirth, whilst advocating the respect of their choices and preferences in the care provided to them.

This article draws on data from the international study “Impact of the Covid-19 pandemic on perinatal mental health (Riseup-PPD-COVID-19)” (Identifier: NCT04595123), conducted in 14 countries (Albania, Argentina, Brazil, Bulgaria, Chile, Cyprus, France, Greece, Israel, Malta, Portugal, Spain, Turkey, United Kingdom) by members of the “Perinatal Mental Health and COVID-19 Pandemic Task Force” within the “Research Innovation and Sustainable Pan-European Network in Peripartum Depression Disorder – Riseup-PPD” (COST Action 18,138). To the best of our knowledge, this is the first study that examines pregnant and postpartum women’s experiences during the Covid-19 pandemic in Cyprus.

### Aim

To examine the impact of the COVID-19 pandemic on the experiences, concerns and needs of pregnant and postpartum women in Cyprus.

## Methods

While the design of the complete “Riseup-PPD-COVID-19” study [27] is prospective observational with a baseline assessment and four follow-ups, the data presented here are extracted only from the baseline assessment. The study has been approved by the Cyprus National Bioethics Committee (ΕΕΒΚ ΕΠ 2020.01.Ι26). All data were completely anonymized, in accordance with the Helsinki Declaration of Research with Human Beings. The study population consisted of women during the perinatal period. Women could thus enter the study during pregnancy or at any time during the first 6 months following childbirth. Therefore, the inclusion criteria at baseline were: Pregnant or biological mothers of a child aged 6 months old or younger; 18 years of age or older; living in Cyprus. The exclusion criteria were: Not being currently pregnant, or not being the biological mother of a child aged 6 months or younger; Women younger than 18 years of age; Living in a country that does not participate in the study; Not consenting to participate in the study. The objective of the analysis conducted for this article is to examine the impact of the pandemic on the experiences, concerns and needs of women in the perinatal period in Cyprus. Similarities and differences between women who were either pregnant or postpartum at the time of the baseline assessment will also be examined.

### Measures

The baseline survey consisted of the following measures [27]: OxCGRT: Oxford COVID-19 Government Response Tracker; COPE-IS: Coronavirus Perinatal Experiences - Impact Survey; COPE-IU: Coronavirus Perinatal Experiences Scale - Impact Update; COPE-CF: Coronavirus Perinatal Experiences Scale - Care Follow Up; BSI-18: Brief Symptom Inventory-18; EPDS: Edinburgh Postnatal Depression Scale; GAD-7: Generalized Anxiety Disorder Screener. COPE-IS [28] has been developed by scientists and clinicians with the intent to understand experiences of new and pregnant mothers during the COVID-19 pandemic. The measure includes a set of questions (both close-ended and open-ended) that assesses several areas of impact of the COVID-19 pandemic: perinatal experiences related to the COVID-19 pandemic; exposure and symptoms; financial impact; social support impact; social distancing and activity restrictions; coping and adjustment; emotional impact; physical and mental health history and substance use. A modified version of the Coronavirus Perinatal Experiences - Impact Survey (COPE-IS) [29] has been administered at the baseline assessment. As the COPE-IS was developed in response to the recent COVID-19 outbreak, psychometric information is not yet available. However, it has been used in several studies [30–33].

### Data collection

Participants were reached and recruited mainly through social media (Facebook, Instagram), through local organizations and maternity units in both public and private hospitals, as well as through personal networks of colleagues and acquaintances of the research team members. Data collection was implemented through Qualtrics®. Participants were asked to click on the project website link (https://momsduringcovid.org/cyprus) to be directed to the online questionnaire for Cyprus. The questionnaire was available in both the Greek and English languages. After an overview of the study, participants were asked to confirm the set of eligibility criteria and to provide their consent in order to access the study. The baseline questionnaire was estimated to take approximately 20-25 minutes to complete. The informed consent provided a clear explanation of how anonymity, confidentiality and protection of data would be safeguarded. In order to address any potential changes in the emotional state of participants due to participation in the study, a debriefing procedure was in place; at the end of the survey, a list of relevant up-to-date psychosocial services and resources in Cyprus was provided to the participants. Contact information for the lead research team was also made available in case participants wished to request additional information.

### Data analysis

Survey data were manually checked for accuracy and consistency before analysis. From an initial number of 1172 respondents, we identified and removed 477 invalid records because either the participant indicated erroneous pregnancy duration or their baby was over 6 months of age. All analysis was conducted using records without missing values. The statistical package SPSS v 26.0 was used for these analyses. Descriptive data analysis was performed to report frequencies and percentages for categorical data, means and standard deviations for numerical variables. Inferential statistics were performed using a significance level of 0.05%. Cross tabulations of categorical variables were tested for independence using the Pearson’s χ2 test of independence. Since Pearson’s χ2 test relies on an approximation, Fisher’s exact test was used when more than 20% of cells had expected frequencies smaller than 5. All numerical variables were tested for Normality using the Kolmogorov–Smirnov test of Normality instead of the Shapiro Wilk test of Normality due to the relatively large sample size (*N* = 695). As all Numerical variables failed their Normality test (*p* < 0.001), further inferential analysis used non-parametric tests. The Mann Whitney test of independent groups, was used for examining the differences between two independent groups.

The qualitative analysis of written, unstructured responses provided by participants to open-ended questions of COPE-IS [28, 29] was performed by two researchers trained in qualitative research methods (E.H., A.C.). All responses to open-ended questions were extracted through SPSS into a Word document. Responses were mainly provided in the Greek language with either Greek or Latin characters and ranged from a phrase to a paragraph of text. The inductive thematic analysis approach was used for the analysis of qualitative data, an approach commonly used in both psychology and health and wellbeing scholarship [34, 35]. The answers to the open-ended questions were analyzed simultaneously by the two researchers following the steps proposed by Braun & Clarke [35] (i.e. familiarization with the data, generation of initial codes, search for themes, review and finalization of themes through an iterative approach). The key themes were then compared and discussed by both parties until consensus was reached. Doubts were discussed with another researcher (MD.C.), who is familiar with intrapartum care in the context under investigation. Although individual responses to open-ended questions might have been brief, they nonetheless provided richness and depth in their entirety [36].

## Results

The study population was 695, comprised of 355 pregnant and 340 postpartum women (with infants up to 6 months of age) residing in Cyprus. The demographic characteristics of the pregnant (51%) and postpartum women (49%) are described in Table 1.

**Table 1:** Demographic characteristics

		All participants	Pregnant women	Postpartum women	*p* value
**Age**	18-25	2.1%	1.8%	2.5%	0.056^a^
	26-35	72,2%	77.3%	67.4%	
	36-45	25.7%	20.9%	30.1%	
**Country of Origin**	Cyprus	92.3%	92.1%	92.5%	0.868^b^
	Other	7.7%	7.9%	7.5%	
**Highest level of education**	Secondary school/ High school	4.4%	3.7%	5.1%	0.056^a^
	Partial university studies	6.3%	7.9%	4.7%	
	University studies (undergraduate)	37.8%	41.1%	34.6%	
	Master or Doctorate	50.9%	46.1%	55.5%	
	Other	0.6%	1.2%		
**Relationship status**	Single	0.4%	0.4%	0.4%	0.583^a^
	Partnered/engaged or living as a couple	17.0%	19.1%	15.0%	
	Married	82.0%	79.7%	84.3%	
	Separated or divorced	0.6%	0.8%	0.4%	
**Residence**	Owned (paid in full)	42.6%	40.4%	44.7%	0.536^a^
	Owned (paying mortgage)	21.6%	20.4%	22.8%	
	Rented	27.0%	28.1%	26.0%	
	Living with parents	6.9%	8.9%	4.9%	
	Living with others	1.2%	0.9%	1.6%	
	Other	0.4%	0.9%		

^a^ based on Pearson’s χ^2^ test of independence

^b^ based on Fisher’s exact test

The mean age of participants was 32.5 years (SD = 4.1). For 60.4% of the participants, this was their first pregnancy (60.6% of pregnant women and 60.3% of postpartum women). Regarding COVID-19 exposure, 1% of the women were diagnosed with the COVID-19 infection, whilst 7% reported having or had symptoms such a cough, fever, breathing difficulties or other symptoms compatible with COVID-19. Four percent of the participants (4%) had been in contact with someone who had been diagnosed with COVID-19 and almost 2% (1.6%) had someone close to them die as a result of COVID-19. The following pre-existing medical conditions were mentioned by a minority of women: respiratory problems (5.3%), mood and/or anxiety disorder (3.6%), a disease compromising the immune system (2.6%), cancer (1.3%), and diabetes (1.2%). In terms of mental health, 2.4% of women reported that they were receiving treatment at the time of the study, 8.1% reported having received treatment in the past, and 3.6% reported having had mental health concerns in the past but have never been treated.

### Impact on life and stress levels

The great majority of the participants (80.9%) interpreted the impact of the pandemic on life as negative (somewhat negative 31.4%, moderately negative 27.4%, extremely negative 22.1%), whilst 12.1% interpreted the impact of the pandemic as positive (slightly positive 6.4%, moderately positive 4.8%, extremely positive 0.9%). For the remaining 7% of the participants, the COVID-19 outbreak had neither a positive nor a negative impact on their life. The mean level of impact on daily life due to the COVID-19 outbreak was 4.39 (SD = 1.53) on a 7-point scale where 1 = not at all and 7 = extremely, with no significant differences between pregnant and postpartum women (M = 4.31, SD = 1.51 and M = 4.46, SD = 1.54 respectively, *p* = 0.300 based on the Mann-Whitney test).

In terms of stress levels, 63.1% reported that their stress levels worsened as a result of the pandemic (worsened significantly 13.8%, worsened moderately 49.3%), whilst one third (34.6%) reported no change. A small percentage of women (2.4%) reported an improvement in their stress levels (improved significantly 0.2%, improved moderately 2.2%). The mean level of stress related to the COVID-19 outbreak was 3.83 (SD = 1.78) and again, no significant differences were found between the pregnant and postpartum women (M = 3.78, SD = 1.73 and M = 3.88, SD = 1.83 respectively, *p* = 0.535 based on the Mann-Whitney test of independent groups, due to the dependent variables not following the normal distribution, *p* < 0.001 in Kolmogorov–Smirnov test of normality).

The major stressors selected by women as “the single greatest source of stress related to the COVID-19 outbreak” at the time of the study were: impact on child, health concerns, financial concerns, impact on vulnerable family members such as elderly parents, and the impact on general well-being due to social distancing and/or quarantine (Table 2). Impact on society (2.9%), community (0.6%), partner (0.9%) or close friends (0.6%) were the least common responses, along with limited access to mental health care (0.4%).

**Table 2:** Single greatest source of stress related to the COVID-19 outbreak

	All participants	Pregnant women	Postpartum women	
	%	%	%	*p* value
Impact on child	23.5	23	24	0.764^a^
Health concerns	20.2	17.5	22.9	0.106^a^
Financial concerns	17.8	19	16.7	0.484^a^
Impact on family members (e.g. elderly parents)	14.5	17.1	12	0.106^a^
General well-being due to social distancing and/or quarantine	9.9	10.4	9.5	0.689^a^

^a^ Based on the adjusted Residuals and a two tail hypothesis

Table 3 presents the overall levels of distress experienced by the women surveyed about (a) own COVID-19 related symptoms or potential illness, (b) COVID-19 related symptoms or potential illness in friends and family, (c) employment and financial impact, and (d) disruptions to social support. No significant differences were found between pregnant and postpartum women. The most common ways to cope with pandemic-related stress were: talking with friends and family (54.2%), limiting the time spent on following news coverage (33.8%), and increasing time on social media (Facebook, Instagram and other) (27.1%). Only 2.4% of the participants considered talking with a mental health professional (e.g. therapist, psychologist, counselor) as a way of coping with stress. Using over-the-counter sleep aids, tobacco or alcohol as a way of coping with stress was also uncommon (0.9, 2.2 and 2.2% respectively).

**Table 3:** Overall levels of distress

Levels of distress (1 = no distress, 7 = highly distressing	All participants	Pregnant women	Postpartum women	
	M(SD)	M(SD)	M(SD)	*p* value*
Own COVID-19 related symptoms or potential illness	4.02 (2.17)	4.05 (2.16)	4.00 (2.18)	0.756
COVID-19 related symptoms or potential illness in friends and family	4.96 (1.89)	4.92 (1.89)	4.99 (1.89)	0.595
Employment and financial impact	5.39 (1.73)	5.4 (1.74)	5.38 (1.73)	0.829
Disruptions to social support	4.23 (2.02)	4.23 (1.97)	4.24 (2.07)	0.923

*based on the Mann Whitney test of independent groups, due to the dependent variables not following the Normal Distribution (*p* < 0.001 in Kolmogorov–Smirnov test of Normality)

### Perinatal care experiences

The most commonly reported changes that pregnant women (*N* = 355) experienced in maternity care as a result of the COVID-19 outbreak were: changed format of maternity care (i.e. no perinatal group classes) (20.6%), cancellation of or reduction in the frequency of maternity visits (16.6%) and a change in the selected maternity department (12.1%). For postpartum women (*N* = 340), the most common change was the ban of support people (e.g. partner, family) during childbirth (47.1%) and the transition from elective vaginal birth to induction or C-section (10%). In terms of changes in postnatal care, the most common responses were the prohibition of visits of family and friends (92.4%), lack of access to breastfeeding consultations or other postnatal support following discharge from hospital (22.9%), lack of information or support for “baby blues” or other issues related to mood (18.5%), and cancellation of postpartum visits (9.7%).

### Pregnant Women’s concerns: support and involvement of family and friends, Child’s health, and intrapartum care

In terms of pregnancy or birth-related concerns in the face of the COVID-19 outbreak, 74.1% of the pregnant women expressed concern about changes in the support and involvement of family and friends at birth, 57.2% about their child’s health, and 43.1% about changes related to medical care during birth. The mean values were calculated on a 7-point Likert scale where 1 = No concern and 7 = Highly concerning were 5.37 (SD = 1.52), 5.82 (SD = 1.32), and 4.92 (SD = 1.77) respectively.

The main concern of most women regarding changes in the support and involvement of family and friends was that they could not have their partner with them during labour:

*“It is understandable and expected that visits will be limited, but at least I would like to ensure that my husband will be present and I don’t even want to think about the possibility of giving birth entirely alone” (*Participant #44*).*

The concern or disappointment of women about the presence of other family members (e.g. older children and parents) during birth or immediately after birth was also a major theme in the women’s responses:

*“It is hard not to have the loved ones next to you and especially my other children not to be able to visit us (*Participant #165*)”*

*“Not even the grandparents of the baby will be allowed to visit [during the hospital stay], stress and anxiety due to self-isolation at home as we will not be accepting visits even from the closest relatives* (Participant #379)”

Many women were worried about the lack of emotional support,

*“To be alone during the times that I need support”* (Participant #278),

as well as about the lack of material assistance at home:

*“Isolation at home, without visits and without practical advice about the care of the newborn” (*Participant *#659).*

Overall, the phrase *“I will be alone/ lonely”* was very commonly brought up.

Women’s concerns about their child’s health revolved mainly around the idea of infection of the fetus or the baby either because of the mother or others:

*“Especially during the first days, I will be very afraid to go out with the baby and I wouldn’t want friends and family members to kiss it”* (Participant #450).

Many women expressed concerns about the consequences of the virus on their baby’s health:

*“In case I get it, how will it affect the health of the baby [fetus]? In case the baby gets it after birth, how much will it [the baby] suffer and what are the consequences on the baby’s health?”* (Participant #609)

*“We hear so much, I don’t know what is right, what is reliable or not”* (Participant #648).

Some women are also worried about the quality of care that will be provided to their baby in case the baby is tested positive, but also about the transmission of the virus within the healthcare setting:

*“[I worry about the baby] getting infected due to carelessness of the clinic’s staff, or due to insufficient disinfection of the place.” (*Participant #497*)*

*“[I worry] that the people in the maternity ward (midwives, doctor) who will be in contact with my baby might have the virus. It upsets me that I have to get tested before I enter the place [ward] – will they get tested before coming in contact with me and my baby since they come in contact with so many people daily?”* (Participant #520)

Concerns about changes related to intrapartum care involved: permission for the partner or any other family member to be present; potential changes in the chosen place of birth including mode of birth and/ or healthcare personnel; the overall quality of care provided; the protocols and the procedures to be followed in case they are positive. Several women were worried that in case they become infected with Covid-19, they should “transfer” and give birth in a designated public hospital with personnel and conditions that were unknown to them:

*“If I am positive I must change my doctor and give birth in a public hospital with a doctor that I have never met before”* (Participant #358).

Other women were also concerned about a potential change in their preferred mode of birth, and more specifically about being required to transition from vaginal birth to a caesarean section:

*“I am afraid they may try to force me to go through a caesarean section”* (Participant #432).

Many women were concerned about the overall quality of care that they would receive, due to the novelty of the virus.

*“If there is a complication due to covid that is new to the doctors and doctors do not know how to react”* (Participant #312),

as well as from the upheaval in the healthcare system and the potentially inadequate response of midwives and doctors:

*“I worry that I will be discharged sooner than normal”* (Participant #450)

*“[Healthcare professionals] not so meticulous and give the due attention”* (Participant #405)

*“Not so much attention to the needs of the mother and the baby”* (Participant #278)

Such concerns were not unfounded, as, indeed, the experiences of some postpartum women illustrate how the pandemic influenced the quality of care, at least during the first wave:

*“Because of the coronavirus, for quite some time during labor, I didn’t have any help from the midwives because they didn’t have enough uniforms to wear and to be with me in the room. They were watching standing outside the door.”* (Participant #565)

*“The midwives were very careless because there were several births at the same time and not enough personnel, resulting in not notifying the doctor on time and not believing me when I was telling them I was having the baby until the baby came out!”* (Participant #127)

Most women who expressed concerns about care during birth mentioned multiple, interrelated worries:

*“I worry in case my husband is not allowed to be with us. Also, due to increased chances [I have], to give birth prematurely and the baby to be transferred to urgent care, I am particularly worried about the decrease of the already few, even before Covid, opportunities to be close to my baby. Another worry is that in the event that I am positive to the virus, the protocol followed, if I am not mistaken, dictates the complete separation of the baby from his/ her mother until the test eventually shows a negative result”* (Participant #636)

*“I worry that due to the virus I will be alone in the maternity ward without my husband. I also worry in case I am tested positive during the prenatal test and I will have to give birth alone, in another city, and also separated from the infant”* (Participant #177)

### Postpartum Women’s concerns: Child’s health and care

In terms of postpartum women’s concerns, 70.3% were concerned about their child’s health (M = 5.97, SD = 1.28 on a 7-point scale, 1 = no concern and 7 = highly concerning). The major concern expressed about the child’s health was whether their child would get infected with the virus. Many women were concerned about both short-term and long-term consequences of COVID-19 on the baby’s health and development. Some of them were concerned because they did not know the consequences of Covid-19 on infants:

*“Will it influence his development?” (*Participant *#314)*

*“How serious is this disease and does it have consequences for those who have been infected?” (*Participant *#101)*

Other women were concerned about specific consequences on the baby’s health:

*“There have been cases where infants also developed Kawasaki’s disease after Covid. Perhaps we don’t know exactly what is happening with this specific virus, the age of infants [its relation to age] and the dangerousness of the combination of the two” (*Participant *#86)*

*“[I worry] that the baby will develop an autoimmune disease as a result of the virus” (*Participant *#119)*

The women were also concerned about the nature of the treatment in case of infection; whether, for example, hospitalization would be necessary, and if so, they were curious to know about the appropriate treatment. They were also concerned about the possible side effects and their consequences, and generally, how the virus will be managed:

*“If it [the baby] gets sick, what will the treatment be and will I be able to care for it?”* (Participant #28)

Other women were mainly concerned about the effects of the government-imposed measures on the psychosocial development of the baby, both in the short term and the long term:

*“[I am concerned about] the limitations in socialization and development activities* e.g. *stimulation from nature, playgrounds, swimming* etc. *(*Participant *#*14*)*

*“How will my child develop in a world where everyone wears a mask and everyone is afraid of other people?” (*Participant *#418)*

Almost half of the postpartum women (49.1%) expressed concern about how they are going to care for their baby because of changes as a result of the COVID-19 outbreak (M = 5.45, SD = 1.5, 1 = no concern and 7 = highly concerning). Such concerns mainly involved the practical considerations of being infected, as well as the availability and safety of childcare:

*“In case I get covid, who is going to take care of my baby and for how long do I have to stay in quarantine?”* (Participant #401)

*“[I am worried] whether I will have easy access to the pediatrician in case something happens to the baby”* (Participant #449)

*“[My concerns] mainly involve the case of quarantine/ separation from the baby and the forced cessation of breastfeeding due to the current protocols in Cyprus*” (Participant #336)

Taking into account that the duration of maternity leave allowance in Cyprus is 18 weeks, many women were worried about trusting the care of their child at such unprecedented times, when they would have to return to work:

*“Where will I leave the baby when I return to work? Do I trust the nursery school? Will the rest of the parents be as careful as I am?”* (Participant #688)

*“I worry that we will be locked inside the house again and the grandmothers will not be able to help [with the care of my baby girl] so that I will be able to work. I worry that we will not be comfortable financially to meet her needs, since the nature of my work is such that in case of a lockdown it will be terminated”* (Participant #408)

### Women’s needs during the perinatal period

The majority of pregnant women felt they were “very well supported” by their primary maternity care provider (78.6%). Similarly, the majority of postpartum women felt they were “very well supported” both by their prenatal (84.7%) and by their postnatal care provider(s) (75%). Nonetheless, several needs emerged when the women were asked about services or resources that could help women and their families cope during the COVID-19 outbreak. Pregnant women rated the following as “very important”: provision of information about COVID and the newborn’s health (78.6%), rapid response to questions and concerns by healthcare providers (73.5%), more one-on-one conversations with their maternity care provider (66.2%), and information about how to reduce stress (58.6%). Items rated primarily as “somewhat important” were: access to a mental health provider (45.4%), interaction with other pregnant women (43.7%), examples of how other women are planning for potential changes in their pregnancy, birth and postpartum care (43.7%), and online support groups (40%).

Postpartum women rated the following as “very important” to help them and their families: information about COVID-19 and infant/child health (88.2%), more one-on-one conversations with their paediatrician (82.9%), rapid response to questions and concerns (77.4%), more one-on-one conversations with their own medical provider (70.3%) and information about how to reduce stress (61.2%). The most common items selected as “somewhat important” were: interaction with other parents (44.4%) and online support groups (38.2%). Importantly, some women brought up the lack of community midwifery in Cyprus and emphasized the need for such a structure, especially during a crisis such as the current pandemic. The women explained that several services offered by community midwifery would be valuable for them, including home visits for education and empowerment during the process of labor, monitoring of the newborn, care for the new mother and provision of breastfeeding advice. A woman commented on the legal restrictions for home births:

*“The state [should] support homebirth. In a low-risk pregnancy, there is no reason during a pandemic for a pregnant woman and a newborn to be exposed to the risks that exist in hospitals and clinics”* (Participant #432)

## Discussion

The present study investigated pregnant and postpartum women’s experiences, concerns and needs during the outbreak of the COVID-19 pandemic in Cyprus. This is the first study in the scientific literature reporting on the impact of the COVID-19 pandemic on the perinatal experiences of a large sample of women living in Cyprus. The results of this study were derived from 695 pregnant and postpartum women. Although the data were collected from an online convenience sample with inherent bias potential, the size of the sample^1^ enables the portrayal of an accurate account of women’s perinatal experiences in Cyprus during the outbreak of the pandemic, in this study.

Consistent with international literature, the results highlight the pandemic’s negative impact on women’s perinatal experiences and mental health [37–42]. The overwhelming majority (80.9%) of the women who participated in this survey reported that the COVID-19 outbreak had an overall negative impact on their life, with almost two thirds of women reporting increased stress levels. A similar study was published by Meaney et al. [38], using quantitative analysis and qualitative content analysis, conducted via social media with 573 pregnant women participating, from America, UK and Ireland. This study found similar results reporting that as a consequence of the COVID-19 pandemic response, women may be affected by increased stress associated with pregnancy because they lack access to antenatal care and perceive that there is less social support as a result of the restrictions implemented. Despite the significant cultural differences between the populations in Meaney’s study and this study, both conclude that it is important to provide supportive care to women during the perinatal period, in all aspects, as they perceive themselves as more vulnerable in pandemic situations.

The three most common reasons for increased stress in relation to the COVID-19 outbreak, reported in this study, were the potential impact of the pandemic on their child, on their own health, and on their finances. For both pregnant and postpartum women, the highest levels of distress were related to employment and financial impact, followed by COVID-19 related symptoms or potential illness of friends and family, and disruptions to social support. Financial strain is, indeed, reported as a major concern for many women in the perinatal period during the pandemic [10, 29, 43]. In contrast to a similar study by Meany et al. [38], it is surprising that only a small minority (2.4%) of the 695 women considered talking with a mental health professional as a way of coping with stress. Meany’s survey found that 92.8% of the 573 women who responded reported using 69 different strategies to reduce stress during the pandemic. In Cyprus, the women tended to cope with pandemic-related stress, first and foremost, by talking with friends and family, limiting time spent on following news coverage and increasing time spent on social media. These findings support the well-documented importance of social networking for women’s well-being during the perinatal period [44–46].

Connecting with family and friends was an important stress coping mechanism for the women. It is not surprising that the most common concern of pregnant women in this study was, by far, whether their partner or other family member would be permitted to be present during birth. This was a valid concern as almost half of the women in the postpartum group (47.1%) gave birth without the support of their partner or a loved one. The absence of birth companions had negative psychological and practical consequences on women’s birth experience [47]. Apparently, restrictions and measures to manage the pandemic were often imposed, without taking into consideration WHO’s [48] guidelines for specific antenatal and intrapartum practices that optimize the physical and psychosocial well-being of mother, baby, and the family in the short and longer term. Feelings of anxiety and uncertainty are inevitable during such sudden and imposed obstetric and public health changes [40].

Overall, being pregnant and giving birth during the COVID-19 pandemic is associated with additional challenges and stressors, depending on context-specific restrictions and measures [10, 49]. The lack of uniform guidelines pertaining to perinatal care during the pandemic, led to significant variability in management across the world [50]. In many cases, the care provided had largely worsened because of the pandemic [51]. In Cyprus, although the overwhelming majority of pregnant women (78.6%) felt they were “very well supported” by their primary maternity care provider, almost half (43.1%) of them were significantly concerned about changes that could potentially occur with connection to intrapartum care. Such concerns included potential changes in the chosen place and mode of birth, the overall quality of care that would be provided, and the protocols and procedures to be followed in case they tested positive. For example, many women were worried that midwives and doctors would not be able to provide quality care either due to practical reasons (i.e. lack of resources in the healthcare system) or due the lack of knowledge on how to manage potential complications arising from COVID-19. Several women were worried about a potential, sudden, transition to the designated public hospital in case they would become infected with the virus, where they would give birth with personnel and conditions that would be unknown to them. Some women were concerned about a potential change in their preferred mode of birth, and more specifically about being required to transition from vaginal birth to a caesarean section. This concern was not unjustifiable as, indeed, 10% of the postpartum women reported having been forced to change their birth plans due to the lack of a coherent response by healthcare professionals and policymakers in the area of perinatal health.

Akin to the pregnant women’s satisfaction levels in terms of care provided to them, the majority of postpartum women felt they were “very well supported” both by their prenatal and by their postnatal care provider(s). Several issues, however, were arising following discharge from the hospital. For example, some women commented on the lack of information or support for “baby blues” or other issues related to mood. For other women, the lack of access to breastfeeding consultations or other postnatal support had a significant effect on their perceived ability to care for their baby and consequently, on their overall well-being. Giving birth during the pandemic brought additional worries [10]; one of the major concerns of postpartum women was about how care for their baby would be affected in the middle of the COVID-19 outbreak. Such worries involved, not only the risk of infection, but also the lack of practical and psychological support from women’s support networks, due to the imposed restrictions and measures for the containment of the virus.

For both pregnant and postpartum women who participated in this study, one of the most common concerns was the potential impact of the pandemic and the virus itself on their child’s health [45, 52]. Pregnant and postpartum women had similar concerns about child’s health. The infection of fetus or baby either through the mother or others was a major reason for worrying. The women also expressed concerns about the quality of care that would be provided in case their baby was tested positive; questions about the nature of the treatment, possible hospitalization, medication and side effects, and generally about the management of the virus were commonly voiced. Questions about both short-term and long-term consequences of the virus on their baby’s health and development were also raised. Postpartum women were particularly concerned about the impact of social distancing measures on their child’s psychosocial development and long-term mental health. The women’s questions often remained unanswered as the provision of reliable and trustworthy information by healthcare professionals was missing. The plethora of conflicting or false information on social media added to the women’s confusion and distress.

### Practical implications

Many pregnant and postpartum women mentioned that the establishment of Community Midwifery is a necessity in Cyprus. This result is congruent with previous research studies in Cyprus and justified that pregnant and postpartum women constantly ask for better perinatal care in the form of community midwifery. One of the major problems in perinatal care in Cyprus is that new mothers and parents do not have access to practical support, monitoring, and care tailored to their needs after discharge from the hospital [24, 25], which is imperative for the health and well-being of the mother, child, and family [48, 53]. Prior to the pandemic, new mothers had to rely primarily on family (e.g. siblings, parents and sometimes grandparents) for practical support, but this was no longer an option due to pandemic-associated restrictions. Community midwifery could also address the reported changes in antenatal care due to the COVID-19 outbreak, including the lack of perinatal classes and the cancellation or reduction of maternity visits. Such a structure could also serve as a reliable source of information addressing the questions reported in this survey and the uncertainty related to the COVID-19 outbreak.

Perinatal health literacy is particularly important in a highly medicalized and decentralized birth environment with high caesarean and low breastfeeding rates as is the case in Cyprus. Despite its crucial role for mother-child well-being, perinatal health literacy in Cyprus has been questioned. The creation of “Baby Buddy Forward” app in 2020, a digital resource of reliable information and evidence-based practices, provides opportunities for enhancing health literacy and reducing related social disparities in Cyprus [54]. The development of other evidence-based eHealth resources and interventions could address pregnant and postpartum women’s concerns and needs in times of crises, such as the COVID-19 pandemic, as part of the perinatal care offered to them [55].

Our findings indicate that social networking is important for women in the perinatal period. However, in times when the imposition of social distancing is paramount to the health of these women, it is important to explore other ways of effective communication, such as virtual services or virtual meetings, involving a network of people and professionals who can add a positive view to the experiences of these women. Furthermore, there should be an exploration of software applications, such as virtual reality environments or guided meditation, that can help pregnant women admitted for labor or postpartum care in hospitals reduce their stress. The use of video teleconferencing during labor and postpartum may offer some support to these women as it may help alleviate their concerns related to the limited hospital visit allowance of family members.

## Conclusion

Pregnant and postpartum women have particular concerns and needs as a result of the current pandemic. This article constitutes a well of scientific knowledge that can be utilized to mitigate the negative consequences of COVID-19 on perinatal experiences, to develop and put into effect plans for the improvement of perinatal care during periods of crisis, as well as to inform policy-making and future decisions on government- imposed restrictions and measures for the management of public health threats. Community midwifery could be a vital source for the improvement of care offered to pregnant and postpartum women, as well as for the prevention of perceived stress-related issues and vulnerability during the challenging times of COVID-19 pandemic. Additional research assessing pregnant and postpartum women’s mental health with validated psychometric tools will add invaluable insight to the findings of this study and contribute towards the implementation of a specialized support system in perinatal care, within the healthcare system in Cyprus.

## Data Availability

The datasets used during the current study are available from the corresponding author upon reasonable request.

## Abbreviations

COVID-19: Coronavirus Disease 2019
WHO: World Health Organization
SHSO: State Health Services Organization
C-section: Cesarean section

## Footnotes

1 In 2019, the number of births in the Government-controlled area was 9548, with a crude birth rate of 10,8 per 1.000 population (Statistical Service of Cyprus, 2020).
